# Characterizing evolving frameworks: issues from Esmail et al. (2020) review

**DOI:** 10.1186/s13012-020-01009-8

**Published:** 2020-07-02

**Authors:** Russell E. Glasgow, Paul A. Estabrooks, Marcia G. Ory

**Affiliations:** 1 Family Medicine, University of Colorado School of Medicine, 13199 E. Montview Blvd, Aurora, CO 80045 USA; 2 Dissemination and Implementation Science Program of ACCORDS (Adult and Child Consortium for Health, University of Colorado School of Medicine, 13199 E. Montview Blvd, Aurora, CO 80045 USA; 3 College of Public Health,Department of Health Promotion, University of Nebraska Medical Center, 986075 Nebraska Medical Center, Omaha, NE 68198-6075 USA; 4 Environmental and Occupational Health, School of Public Health, Texas A&M University, SPH Administration Building, 212 Adriance Lab Rd., TAMU Bldg #1518 | TAMU MS 1266, College Station, TX 77843 USA; 5 Center for Population Health and Aging, Texas A&M University, SPH Administration Building, 212 Adriance Lab Rd., TAMU Bldg #1518 | TAMU MS 1266, College Station, TX 77843 USA; 6 Strategic Partnerships and Initiatives, Texas A&M Health, Texas A&M University, SPH Administration Building, 212 Adriance Lab Rd., TAMU Bldg #1518 | TAMU MS 1266, College Station, TX 77843 USA

**Keywords:** Review, RE-AIM, Implementation science framework, Classification

## Abstract

There are complex issues in understanding and categorizing implementation science theories, models, and frameworks. Systematic reviews of these models are important undertakings for synthesizing current knowledge. The issues involved are even more challenging when reviewing a large number of frameworks and when some of the frameworks have evolved significantly over time. This paper addresses how the RE-AIM (Reach, Effectiveness, Adoption, Implementation, and Maintenance) framework was described in the recent Esmail (2020) review and identifies four mischaracterizations. This is followed by a more general discussion of how advances or extensions of frameworks after an original source publication or influential review tend to be overlooked. We discuss why inadvertent mischaracterization of what a framework is and is not, and what it can and cannot be used for, can have deleterious consequences. Finally, we suggest initial ideas about what could be done to prevent or alleviate some of these problems by reviewers, framework developers, and scholars at large.

We are writing concerning the recent review article by Esmail et al. “A scoping review of full-spectrum knowledge translation theories, models, and frameworks” in *Implementation Science* [1]. While we see this article as making an important contribution to the literature, it also, likely inadvertently, mischaracterized the RE-AIM framework in a few salient areas. Our intent is to provide some clarity on these areas, highlight the implications of mischaracterizations of theories, models, and frameworks (TMF), and offer ideas for positive solutions that can advance implementation science.

It is important to start with the potential source of mischaracterizations related to the RE-AIM framework. The only RE-AIM reference cited in the review article was the original 1999 citation [2]. The RE-AIM framework like many others has evolved considerably over the past 20 years. Since 1999, we have published several reviews, updates, modifications, newer applications, and guidance for use of RE-AIM [3, 4]. In brief, four of the statements made about RE-AIM are incorrect, some were partially correct up until 2013, but others have not been accurate for the past decade or more. Below, we summarize the mischaracterizations, followed by a more accurate statement about the RE-AIM framework:

1. *The RE-AIM framework is solely quantitative in nature*. There have been reports of qualitative uses of RE-AIM for at least a decade. Qualitative assessments of reasons why RE-AIM results were found have been recommended in published reviews since at least 2013 and explicitly called out as a strong recommendation in Kessler et al. [5].
2. *RE-AIM assumes all dimensions are equally important*. All RE-AIM dimensions are important and contribute to overall public health impact. However, since 2006 [6], we have discussed pros and cons of different weightings of RE-AIM elements. We advise users to consider all elements, but to prioritize the dimensions most important to stakeholders in a given project [7].
3. *The time interval for assessing implementation and maintenance are arbitrary*. The time interval for implementation and maintenance are not arbitrarily mandated to be 6 months and 2 years, respectively. In our first publication on RE-AIM in 1999 [2], this was the case, but in many articles since then, the framework and these temporal perspectives have evolved—e.g., from sustained implementation directly after grant funding ceases to as long as 5 years after the initial implementation was completed [8].
4. *The RE-AIM framework is only an evaluation framework*. RE-AIM is not just an evaluation framework. It has been widely and successfully used to plan interventions for almost 15 years [9] and, more recently, to help guide adaptations during implementation. In addition, the PRISM framework which includes RE-AIM was added in 2008 [10] to address contextual factors that impact RE-AIM outcomes.

What promoted this letter is that similar mis-categorization and statements that RE-AIM is only an evaluation framework, cannot be used for any other purpose, is not an implementation model, or cannot be used as a qualitative approach are *often heard from grant and journal article reviewers despite abundant evidence to the contrary*. These inaccurate assertions have had negative impacts on advancing science and on the evaluation of proposals of several promising emerging researchers.

We take partial responsibility for this situation, since our group has not consistently provided clear guidance or unambiguous statements on these issues. Although we try to keep our website [3] current, there have been inconsistencies on the website, and as discussed above, the model has and continues to evolve. We have recently published the Glasgow et al. [4] paper to more clearly describe what is now Expanded RE-AIM/PRISM, the meaning and distinctions among various RE-AIM dimensions, and provide guidance on related issues.

Our larger concern is that similar mischaracterization issues apply not only to RE-AIM, but also to other TMFs. From discussions with other TMF developers, we speculate that many reviewers and scientists rely only on either (a) the original TMF article without considering refinements and extensions over time or (b) an influential review, or even increasingly reviews of reviews. We understand that when reviewing the broad range of TMFs, it is not possible to review the entire literature on each TMF, but conclusions in major publications have consequences.

Beyond specific issues concerning RE-AIM, we would like to begin discussion of how the field can collectively prevent or address unintended consequences of earlier categorizations or initial TMF papers being cited as conclusive without considering additional literature. We recommend that discussion sections of reviews include a highlighted statement that not all studies on each TMF were evaluated, that there may have been changes since an original publication on a given TMF, and that readers should not rely solely on that review and/or the original reference. We welcome other suggestions on what reviewers, TMF developers, and readers can do to alleviate this troubling and relatively common issue.

## Data Availability

N/A

## Abbreviations

RE-AIM: Reach, Effectiveness, Adoption, Implementation, and Maintenance
TMF: Theories, models, and frameworks
PRISM: Practical, Robust, Implementation, and Sustainability Model
